# Extending the Privacy Calculus to the mHealth Domain: Survey Study on the Intention to Use mHealth Apps in Germany

**DOI:** 10.2196/45503

**Published:** 2023-08-16

**Authors:** Niklas von Kalckreuth, Markus A Feufel

**Affiliations:** 1 Division of Ergonomics Department of Psychology and Ergonomics (IPA) Technische Universität Berlin Berlin Germany

**Keywords:** mHealth, mobile health, confidential, privacy calculus, privacy, intention to use, adoption, data autonomy, social norms, trust in the provider, trust, privacy concern, benefit, attitude to privacy, survey, intention

## Abstract

**Background:**

With the increasing digitalization of the health sector, more and more mobile health (mHealth) apps are coming to the market to continuously collect and process sensitive health data for the benefit of patients and providers. These technologies open up new opportunities to make the health care system more efficient and save costs but also pose potential threats such as loss of data or finances.

**Objective:**

This study aims to present an empirical review and adaptation of the extended privacy calculus model to the mHealth domain and to understand what factors influence the intended usage of mHealth technologies.

**Methods:**

A survey study was conducted to empirically validate our model, using a case vignette as cover story. Data were collected from 250 German participants and analyzed using a covariance-based structural equation model.

**Results:**

The model explains R2=79.3% of the variance in intention to use. The 3 main factors (social norms, attitude to privacy, and perceived control over personal data) influenced the intention to use mHealth apps, albeit partially indirectly. The intention to use mHealth apps is driven by the perceived benefits of the technology, trust in the provider, and social norms. Privacy concerns have no bearing on the intention to use. The attitude to privacy has a large inhibiting effect on perceived benefits, as well as on trust in the provider. Perceived control over personal data clearly dispels privacy concerns and supports the relationship of trust between the user and the provider.

**Conclusions:**

Based on the privacy calculus, our domain-specific model explains the intention to use mHealth apps better than previous, more general models. The findings allow health care providers to improve their products and to increase usage by targeting specific user groups.

## Introduction

### Background

The use of digital health products, which promise to increase the effectiveness and efficiency of health care delivery, is on the rise. Between autumn 2019 and summer 2021, downloads of mobile health (mHealth) apps in Germany doubled to 2.4 million [1]. mHealth apps run on mobile devices and may provide medical services ranging from individual care to public health measures [2]. They are said to improve individual health competence and, ultimately, motivate users to deal with their own health more responsibly through interventions and access to information, simplified communication with experts, and the tracking of health data [3-5]. In addition to these advantages, there are also risks associated with using mHealth apps. For example, the security infrastructure of many apps is currently inadequate and does not meet the requirements for protecting user data (eg, the General Data Protection Regulation [GDPR] in the European Union and the Health Insurance Portability and Accountability Act [HIPAA] in the United States) [6]. It is therefore not surprising that mHealth users are becoming increasingly sensitive to data privacy and data security [7-9]. Given the pros and cons of using mHealth technologies, it is essential to take a close look at the factors that influence users’ intention to (not) use them in order to inform and improve mHealth technology design and, ultimately, increase the uptake of safe and efficient technologies. To examine why people intend (not) to use mHealth apps, we decided to build on the privacy calculus model.

In this study, we focus on the use of health insurance apps because, on the one hand, there is already a large number of users and, on the other hand, a large number of potential users due to the mandatory membership in a health insurance company in Germany [1].

### Related Work

The privacy calculus model originally postulated that users of social network sites (SNSs) perform a calculus between the expected loss of privacy and the potential gain of disclosure when deciding whether to use it [10]. That is, the model suggests that people compare potential benefits and costs to calibrate their intention to use the SNS technology [11-13]. If the sum of the drivers (benefits) is greater than that of the inhibitors (costs), people will use the technology. If the number of inhibitors is greater, the use of the technology is rejected [11,14,15]. The privacy calculus model was successfully used to predict the intention to use SNSs [16] and e-commerce websites [17]. Based on the privacy calculus model, we aim to understand which factors have a concrete influence on the cost-benefit calculation underlying the intention to use mHealth apps.

Thus far, 3 studies that have examined the intention to use mHealth apps based on the privacy calculus model. They were limited either by the lack of explained variance (*R*^2^ values did not exceed 0.5 [11,18] or were not reported [19]) or marginal model fit values [20], which indicate that the used model did not properly fit the observed data [19]. Conceptually, we think these studies [11,18,19] underrepresented the following 3 domain-specific factors influencing the intention to use mHealth technologies:

- When examining the intention to use mHealth technology, the data autonomy granted to the users, that is, the control over granular privacy settings to limit access to their data [14,16], was not taken into account [18,19] or only partially accounted for via the concepts of privacy concerns [11]. Studies have shown, however, that data autonomy influences the intention to use data-collecting mHealth technology [21].
- Although the direct or indirect influence of trust in the provider on the intention to use mHealth technology has been examined in 2 studies [11,19], the individual’s interest in the object represented in the trusting relationship—here the protection of personal data—has not been considered [22]. If the user is not interested in the security of personal data, a relationship of trust concerning the use of data would be irrelevant. Consequently, to be able to make statements about a trusting relationship, the general attitude to privacy should be considered [22,23].
- None of the existing studies considered the influence of social norms, such as social pressure from family and friends. However, there is evidence that social norms influence the acceptance of mHealth technology for disease prevention, especially in healthy individuals [24,25].

### Aim of This Study

To achieve our overall goal (ie, to explain the intention to use data-collecting mHealth technology), we address 3 subgoals in this article: (1) we investigate whether perceived data autonomy reduces privacy concerns and has a positive effect on the intention to use mHealth apps, (2) we explore the influence of an attitude to privacy on trust in the provider, and (3) we examine the influence of social norms on the intention to use mHealth apps. To implement these subgoals, we first explain our model and derive hypotheses. We then validate our model in a survey study using a covariance-based structural equation model (CB-SEM). After discussing the results, we derive theoretical and practical implications and reflect on the limitations of the study. We end our paper with a conclusion concerning our objectives.

### Model Description and Hypotheses

**Figure 1:**
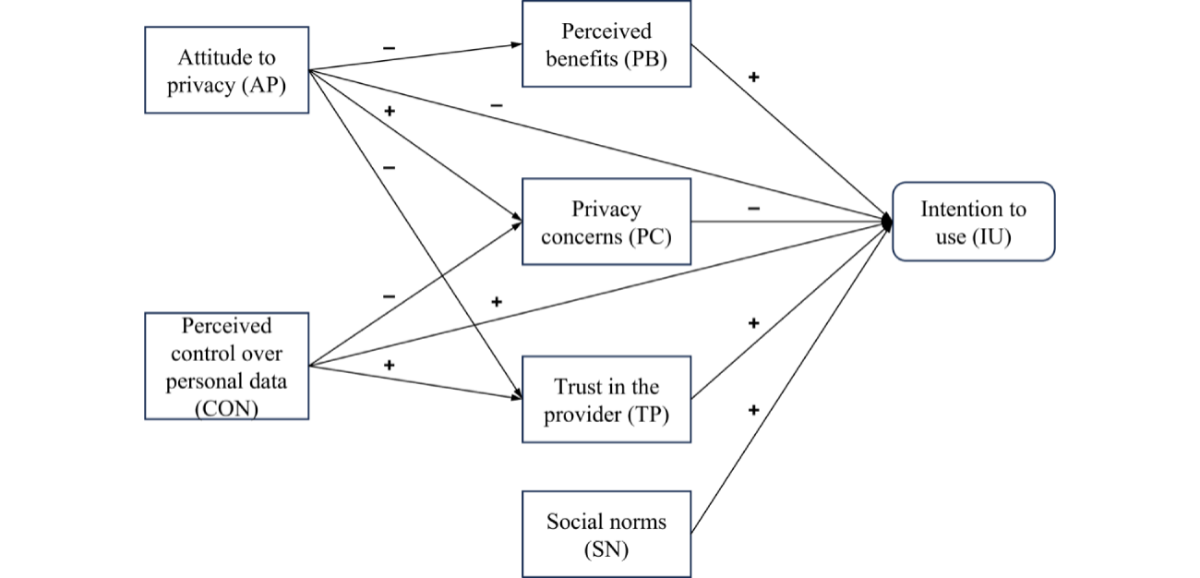
Extension of the privacy calculus model to predict intention to use mHealth apps [22].

To predict and examine the intention to use mHealth apps, we adapted a privacy calculus model from the SNS domain [12]. In contrast to privacy calculus models in the mHealth area, in the SNS domain it is common to examine the influence of social norms and perceived data autonomy. Therefore, in addition to the constructs of perceived benefits, privacy concerns, and trust in the provider, the adapted model also included the constructs of perceived control over personal data (subgoal 1) and social norms (subgoal 3) [12]. Finally, we added the attitude to privacy to the model to cover subgoal 2 from above. Unlike previous studies [11,18,19], we refrained from adding health-specific factors (eg, health concerns) to reduce the complexity and increase general applicability of the model. Figure 1 shows the final model with drivers (+) and inhibitors (–), which we will elaborate on in turn.

### Perceived Benefits

Perceived benefits are both the hedonistic and the utilitarian reasons people may have to use a product or service. Hedonistic reasons may be that the process of using a technology is fun and enjoyable, irrespective of what may be achieved by using it [16,26]. On the other hand, utilitarian reasons are mainly associated with an increase in productivity and efficiency (eg, time savings, economic advantages) [17,27,28]. In the area of mHealth, utilitarian advantages may also relate to the simplification of treatments and coordination between different medical institutions, which can lead to more efficient treatments and, ultimately, better health outcomes [4,5,11]. There is evidence that the perception of benefits has a driving influence on the intention to use data-collecting and disclosing mHealth information technology [4,21].

- H1: Perceived benefits positively influence users’ intention to use mHealth apps.

### Privacy Concerns

Privacy concerns describe users’ concerns about a possible loss of privacy using web-based apps due to privacy risks, such as data leaks and data misuse [15]. These concerns are driven by situational risk perceptions, for example, data that are not secure with a particular provider [15]. Thus, privacy concerns can be thought of as a situational motivator to be careful when disclosing personal data [14,29,30], and, ultimately, to inhibit the use of health technologies that require disclosure of personal data [21,31,32].

- H2: Privacy concerns negatively influence users’ intention to use mHealth apps.

### Trust in the Provider

Trust is a complexity-reducing variable because it makes the trustor bear a perceived risk when cooperating with a trustee [33]. In other words, trust is a psychological state where a person accepts being vulnerable to the actions of another party because the person expects that the other party will carry out a certain action in their interest, regardless of whether the action is monitored [34,35]. When interacting with information technology, people’s focus is less on trust in the functionality of the system and more on trust in the provider to protect their data and privacy [36,37]. Various studies have shown that trust in the provider has a significant positive influence on the acceptance of mHealth technologies and their intended use [3,38-41].

- H3: Trust in the provider positively influences users' intention to use mHealth apps.

### Social Norms

Social norms are social and psychological factors that are inherent in group dynamics and strongly influence individual human behavior [14]. People tend to behave in ways that are (socially) accepted to continue to benefit from the advantages of being part of a social group (injunctive norms). Violation tends to be punished with disapproval and possibly social ostracism [14,42,43]. Besides, individuals follow the behaviors of others (descriptive norms) [43]. In the case of health prevention through mHealth technology, users’ intention to use mHealth technology is influenced by both the approval of technology use in their social environment (eg, injunctive norms friends and family) and the descriptive norms based on how and when a technology is used in the social environment [12,24,44].

- H4: Social norms positively influence users’ intention to use mHealth apps.

### Perceived Control Over Personal Data

Perceived control is a psychological construct that describes individuals’ perceptions of the extent to which they can influence and control the achievement of a certain goal and the resources that are necessary to do so [11,45]. In the context of mHealth apps, this involves the perceived ability to control which health data are collected and who can access them [11,21,40]. Various studies have shown that if control over personal data is perceived to be limited, privacy concerns increase [8,11,46]. By contrast, if people think that they can control their data, their intention to use mHealth technology [8,11] and their trust in the technology provider increases [11,22,33,40].

- H5a: Perceived control over personal data positively influences users’ intention to use mHealth apps.
- H5b: Perceived control over personal data negatively influences users’ privacy concerns.
- H5c: Perceived control over personal data positively influences users’ trust in the provider.

### Attitude to Privacy

We define the attitude to privacy as a user’s general tendency to consider privacy and data security to be important or a user’s disposition to value privacy [15]. The inclusion of this construct in the privacy calculus model is particularly important in the mHealth context because disclosure of health data tends to be more consequential than data stored on other technologies, such as SNSs [47]. A strong attitude toward data protection has an inhibiting effect on people’s intention to disclose data (ie, their privacy concerns) and their intention to use a data-collecting technology altogether [15,28,48]. Once data have been disclosed, users with a strong attitude to privacy are more interested in the whereabouts of their data and consequently more cautious when it comes to trusting the provider using their private data [22,49-51]. Finally, whereas the perception of potential risks may be overinflated due to strong attitudes to privacy, potential benefits of technology use may be undervalued [52-56].

- H6a: Attitude to privacy negatively influences users’ intention to use mHealth apps.
- H6b: Attitude to privacy positively influences users’ privacy concerns.
- H6c: Attitude to privacy negatively influences users’ trust in the provider.
- H6d: Attitude to privacy negatively influences users’ perceived benefits.

Now that we have explained the theoretical basis of our model, we evaluate the underlying hypotheses in a survey study. In the next section, we describe the methodological basis of this study.

## Methods

### Participants

The theoretical framework described in Figure 1 was empirically tested using data gathered via an online survey that was performed as part of a bigger study in cooperation with a German health insurance company (BARMER), one of the largest and best-known health insurance companies in Germany. The survey was administered by a commercial survey agency in Germany (Norstat GmbH), which also organized the entire survey process (programming the online study and collecting the data). We targeted a sample of at least 250 participants to be able to calculate the model validly [20]. Participants were individuals who registered with Norstat GmbH as survey participants. In addition to being a resident of Germany and a native German speaker, the prerequisites were that the participants were customers of a German health insurance company, as the case vignette centered on a German health insurance app. The minimum age for participation was 18 years, as this is also the minimum age for admission as a Norstat panel member. There were no prerequisites regarding gender. Data collection took place from March 11, 2021, to March 17, 2021. Our estimated minimum time to complete the survey was 5 minutes. This was ensured by the system allowing participants to continue the survey only after a certain amount of time (60 seconds for the consent form, 30 seconds for the case vignette, and 210 seconds for the questionnaire). The mean and median participation times were both 6 minutes with a standard variation of 42 seconds. Participants volunteered to participate after giving informed consent and received compensation (€0.80 [US $0.90]) for taking the survey.

### Ethical Considerations

Because a third party (Norstat GmbH) contacted potential participants and collected the data, we did not have direct contact with participants or access to any personally identifying participant information. We obtained only completely anonymous data. Consequently, we were able to guarantee full anonymity and privacy of the participants, which conforms to the ethical guidelines of the German Research Foundation. Thus, based on the guidelines of the Ethics Committee of our Institute (Institute of Psychology and Ergonomics) no additional ethics board review was mandatory [57].

### Materials

Following a practice that is often used in technology acceptance studies [58], the study used a case vignette to evoke a typical situation where an mHealth app would be used and described the trade-off between the benefits of using it and its data privacy risks. We decided to describe a health insurance app in the case vignette because, as already described, they currently account for the largest share of mHealth app downloads in Germany [1]. In particular, the case vignette (Multimedia Appendix 1) describes a situation in which a friend “Alex” uses the app of his health insurance on a wearable to track his health behavior (ie, physical activity). By participating in the bonus program of this insurance, Alex may receive a bonus of up to €100 (US $112) for working out regularly (a direct benefit), but the insurance may also deny covering treatment costs due to an unhealthy lifestyle (a possible risk). To assess the factors included in the privacy calculus model displayed in Figure 1, we used a 30-item questionnaire (Multimedia Appendix 2; also see [14,16,27,42,53,59-62]), which we developed following the methodology of Moore and Benbasat [63]. All items were measured on a 7-point Likert scale that ranged from 1 (strongly disagree) to 7 (strongly agree).

### Procedure

The survey consisted of 3 parts. In the first part, demographic data of the respondents were recorded, such as age, gender, and educational level. In the second part, the respondents were asked about their individual experience with mHealth apps as well as their current use of wearables such as fitness trackers and smartwatches (also beyond health apps). In the third part, the participants received the case vignette and were asked to answer the questionnaire. The order of the questions in the questionnaire was randomized for each participant.

### Analyses

To test the model outlined in Figure 1, a CB-SEM was used, which is a common approach to theory testing and confirmation [64]. The CB-SEM was carried out with *lavaan* [65] (version 0.6-9; R Foundation) in RStudio (version 1.3.1093; Posit, PBC), using the maximum likelihood estimator. All items of the questionnaire were included in the analysis and restricted to load on the respective constructs described above and in Figure 1.

## Results

### Survey Characteristics

A total of 336 observations were collected. After deleting observations that were unusable because of missing responses, a final sample of 250 observations (126 male and 124 female) was used for further analysis. The mean age of participants was 46.5 years (SD 15.2 years). The demographic characteristics of the sample are summarized in Table 1.

**Table 1:** Demographic data of the sample (N=250).

Demographic characteristic			Frequency, n (%)
**Gender**			
	Male	126 (50.4)	
	Female	124 (49.6)	
**Education**			
	No degree	2 (0.8)	
	School leaving certificate	39 (15.6)	
	Secondary school certificate	88 (35.2)	
	General qualification for university entrance	57 (22.8)	
	University degree (bachelor’s or master’s)	62 (24.8)	
	PhD	1 (0.4)	
	Other	1 (0.4)	
**Experience with mHealth^a^ apps**			
	Regular use of mHealth apps	124 (49.6)	
	Occasional use of mHealth apps	34 (13.6)	
	No use of mHealth apps	92 (36.8)	
**Usage of wearables**			
	Regular use of wearables	73 (29.2)	
	No use of wearables	177 (70.8)	

^a^mHealth: mobile health.

### Assessment of the Structural Model

The internal consistency of the scales as well as convergent validity and discriminant validity of the measured constructs are shown in Tables 2 and 3. Internal consistency was evaluated with Cronbach α with the criterion of α≥.7 [66]. All constructs surpass the recommended value, and therefore internal consistency can be assumed. The convergent validity was assessed following Hair et al [20] using the following 3 criteria: (1) the significance of the factor loadings, which exceed the criterion value of 0.5; (2) the average variance extracted (AVE) should be greater than 0.5; (3) the composite reliability (CR) should surpass the minimum threshold of 0.6. All subscales met these 3 criteria.

Discriminant validity was evaluated by the Fornell-Larcker Criterion [20,67]. For each latent variable, the square root of AVE (diagonal elements) must be larger than the correlation between this latent variable and any other latent variable (off-diagonal elements). As shown in Table 3, this criterion was fulfilled for all latent variables.

To further assess the quality of the structural model, we computed overall measures of goodness of fit, following the recommendations of Hair et al [20], and calculated the model chi-square statistics, the comparative fit index (CFI), and the root-mean-square error of approximation (RMSEA). Specific thresholds for high model complexity (≥30 observed variables) and small sample size (≤250 observations) apply. The fit indices, their values, and the specific threshold values are presented in Table 4.

**Table 2:** Quality criteria of the constructs.

Latent variable and item			Mean (SD)		Standardized factor loading		AVE^a^		CR^b^		Cronbach α
**AP^c^**							0.918		0.957		.961
	AP01	3.34 (1.76)		0.943							
	AP02	3.36 (1.77)		0.972							
**CON^d^**							0.795		0.951		.951
	CON01	4.40 (1.67)		0.885							
	CON02	5.09 (1.60)		0.873							
	CON03	4.94 (1.64)		0.889							
	CON04	4.64 (1.67)		0.923							
	CON06	4.51 (1.68)		0.886							
**IU^e^**							0.806		0.926		.935
	IU01	4.74 (1.86)		0.904							
	IU02	4.68 (1.94)		0.889							
	IU04	4.64 (1.90)		0.902							
**PB^f^**							0.757		0.949		.949
	PB01	4.25 (1.65)		0.838							
	PB03	4.11 (1.62)		0.901							
	PB04	4.31(1.70)		0.883							
	PB05	4.08 (1.73)		0.864							
	PB06	4.01 (1.67)		0.903							
	PB07	3.70 (1.64)		0.827							
**PC^g^**							0.752		0.938		.938
	PC02	2.85 (1.59)		0.877							
	PC07	2.71 (1.46)		0.860							
	PC08	2.76 (1.48)		0.873							
	PC09	3.35 (1.56)		0.832							
	PC10	3.07 (1.59)		0.891							
**SN^h^**							0.782		0.946		.946
	SN01	4.54 (1.72)		0.868							
	SN02	4.52 (1.64)		0.853							
	SN03	4.96 (1.81)		0.875							
	SN04	4.50 (1.78)		0.890							
	SN05	4.63 (1.85)		0.925							
**TP^i^**							0.819		0.948		.947
	TP01	4.13 (1.60)		0.907							
	TP02	4.29 (1.64)		0.889							
	TP03	4.20 (1.74)		0.902							
	TP07	4.30 (1.75)		0.921							

^a^AVE: average variance extracted.

^b^CR: composite reliability.

^c^AP: attitude to privacy.

^d^CON: perceived control over personal data.

^e^IU: intention to use.

^f^PB: perceived benefits.

^g^PC: privacy concerns.

^h^SN: social norm.

^i^TP: trust in the provider.

**Table 3:** Fornell-Larcker Criterion: square root of AVE^a^ and correlation between latent variables (off-diagonal elements).^b^

	AP^c^	CON^d^	IU^e^	PB^f^	PC^g^	SN^h^	TP^i^
AP	*0.958*	—^j^	—	—	—	—	—
CON	–0.767	*0.891*	—	—	—	—	—
IU	–0.781	0.770	*0.898*	—	—	—	—
PB	–0.729	0.560	0.747	*0.870*	—	—	—
PC	0.640	–0.803	–0.660	–0.467	*0.867*	—	—
SN	–0.668	0.753	0.819	0.487	–0.610	*0.883*	—
TP	–0.877	0.851	0.811	0.639	–0.696	0.690	*0.905*

^a^AVE: average variance extracted.

^b^Diagonal elements are in italics.

^c^AP: attitude to privacy.

^d^CON: perceived control over personal data.

^e^IU: intention to use.

^f^PB: perceived benefits.

^g^PC: privacy concerns.

^h^SN: social norm.

^i^TP: trust in the provider.

^j^Not applicable.

**Table 4:** Goodness-of-fit measures of the CB-SEM^a^, following the recommendations for complex models and small samples [20].

Fit indices	Sample	Recommended cutoff criterion
Chi-square (*χ*^2^)	933.148	—^b^
Degrees of freedom (*df*)	391	—
Normed chi-square (*χ*^2^/*df*)	2.387	<3
CFI^c^	0.940	>0.93
RMSEA^d^	0.074	Values < 0.08 with CFI >0.93

^a^CB-SEM: covariance-based structural equation modeling.

^b^Not applicable; they do not have cutoff criteria. Nonetheless, they are part of the fit indices report as standard information, which is needed for the normed chi-square (which has a cutoff).

^c^CFI: comparative fit index.

^d^RMSEA: root-mean-square error of approximation.

All fit indices indicate a good fit. The test of overall model fit resulted in a chi-square value (*χ*^2^) of 933.148 with 391 degrees of freedom (*df*) and a *P* value of <.001. Because of the dependence of the chi-square statistic on sample size and model complexity, the significant *P* value is negligible, and the use of the normed chi-square (*χ*^2^/*df*) is advisable [20]. For our model, this ratio indicates a good fit with *χ*^2^/*df*=2.387, which is below the threshold of 3. Furthermore, an absolute RMSEA and an incremental fit index (CFI) were calculated. Both the RMSEA (0.074) and the CFI (0.94) meet the necessary criteria for a good model fit.

### Results of the Structural Model

**Figure 2:**
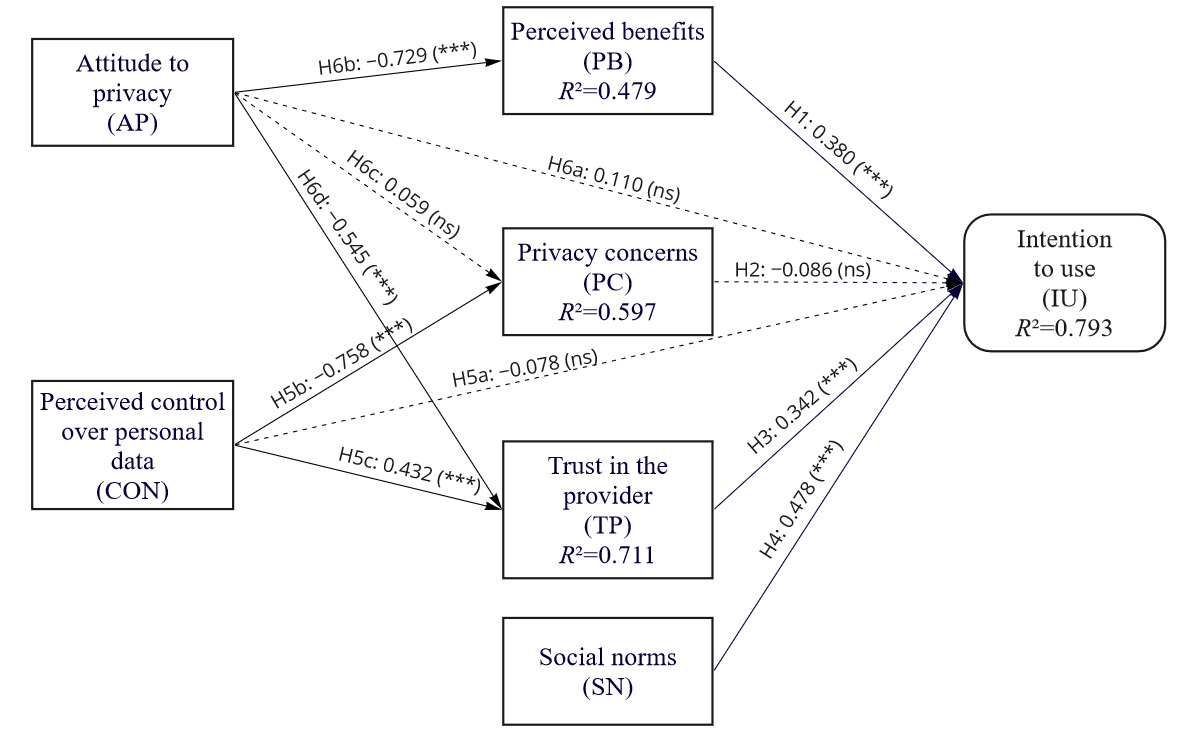
Factor relationships in the structural model. Solid lines represent statistically significant links and dashed lines represent statistically nonsignificant links. *P<.05. **P<.01. ***P<.001. ns: not significant.

After the fit of CB-SEM has been evaluated, we now describe the structural model in more detail. Figure 2 represents the path coefficients and the corresponding *P* values. We include age, gender, education, mHealth experience, and the usage of wearables as control variables to control for the variance explained by these variables.

Table 5 summarizes the detailed analysis of the path coefficients. The *R*^2^ value for the intention to use and the other *R*^2^ values (for perceived benefits, privacy concerns, and trust in the provider) exceed the cutoff value of 0.4 [68] and suggest a good model fit. Consistent with our expectations, perceived benefits has a significant effect on the intention to use (*P*<.001), as well as trust in the provider (*P*<.001) and social norms (*P*<.001), supporting H1, H3, and H4. Privacy concerns do not have a significant effect on the intention to use (*P*=.14). Consequently, H2 is rejected. Perceived control over personal data has significant effects on privacy concerns (*P*<.001) and trust in the provider (*P*<.001), while there is no significant effect on intention to use (*P*=.40). Thus, H5a is rejected, while H5b and H5c are supported. The attitude to privacy has significant effects on perceived benefits (*P*<.001) and trust in the provider (*P*<.001), thus supporting H6b and H6d. The attitude to privacy, however, has no significant effect on the intention to use (*P*=.20) as well as on privacy concerns (*P*=.41), rejecting H6a and H6c. Our model explains *R*^2^=79.3% of the variance in our main dependent variable, that is, intention to use mHealth technologies, controlling for demographic variables and the reported usage of wearables and mHealth apps. The control variables gender (*P*=.75), education (*P*=.92), and the reported usage of wearables (*P*=.24) were not related to the intention to use, whereas age was related negatively (*P*=.002) and the experience with mHealth apps was related positively to intention to use (*P*=.03).

**Table 5:** Path coefficients and hypothesis testing.

Hypothesis	Construct A → B	Path coefficient	*P* value	Supported
H1	PB^a^ → IU^b^	0.380	<.001	Yes
H2	PC^c^ → IU	–0.086	.14	No
H3	TP^d^ → IU	0.342	<.001	Yes
H4	SN^e^ → IU	0.478	<.001	Yes
H5a	CON^f^ → IU	–0.078	.40	No
H5b	CON → PC	–0.758	<.001	Yes
H5c	CON → TP	0.432	<.001	Yes
H6a	AP^g^ → IU	0.110	.20	No
H6b	AP → PB	–0.729	<.001	Yes
H6c	AP → PC	0.059	.41	No
H6d	AP → TP	–0.545	<.001	Yes
Controls	Age → IU	–0.173	.002	N/A^h^
Controls	Gender → IU	–0.02	.75	N/A
Controls	Education → IU	0.006	.92	N/A
Controls	Experience with mHealth^i^ → IU	0.174	.03	N/A
Controls	Wearable usage → IU	0.082	.24	N/A

^a^PB: perceived benefits.

^b^IU: intention to use.

^c^PC: privacy concerns.

^d^TP: trust in the provider.

^e^SN: social norm.

^f^CON: perceived control over personal data.

^g^AP: attitude to privacy.

^h^N/A: not applicable. Controls are not part of the hypothesis section; consequently, there is nothing that could be supported or rejected. Nonetheless, they are part of the results.

^i^mHealth: mobile health.

## Discussion

### Principal Findings

This study examined whether the intention to use mHealth apps could be described by an extended privacy calculus model that considers social norms, perceived data autonomy, and the attitude to privacy of the user. Furthermore, we examined the influence of control variables on intention to use, of which mHealth experience and age had a significant effect. Users who already had experience with mHealth apps and were familiar with similar apps had a greater intention to use them. This has already been demonstrated in other studies [69,70]. Age had a significant inhibiting effect on intention to use, which is in line with other studies on mHealth technology [69,70].

With overall complexity similar to existing models, the suggested model explains the variance (*R*^2^) in users’ intention to use mHealth apps more effectively than other reported models (where values do not exceed 0.5 [11,18] or are not reported [19]).

An important, albeit expected, finding is that the more benefits users perceive, the higher their intention to use mHealth apps. That is, if the product is perceived to be useful or if there are benefits (eg, economic or utilitarian) users value, they are more likely to use it. Unexpectedly, in the context of health insurance apps, perceptions of benefits outweigh perceived risks, which had no part in our privacy calculus. Our model suggests that this can be attributed in part to the level of perceived control over personal data or a lack thereof, which acts as a mitigating factor that reduces or increases users’ perception of risk in the context of data protection (negative path coefficient=–0.758). That is, the more users think they are in control of their data, the less concerned they are about disclosing personal data and vice versa.

The results of this study also underline the salient role of users’ attitudes to privacy. According to the model, the more trust is placed in the provider, the more likely the mHealth app will be used. This relationship is in part explained by the trait-factor attitude toward privacy. When privacy issues are particularly important to users, trust in the provider tends to be lower (negative path coefficient=–0.545). In addition, users’ attitude to privacy has an indirect influence on the intention to use of mHealth apps and wearables. Users’ perceptions of benefits are negatively correlated with the attention they pay to data privacy (negative path coefficient=–0.729). Thus, the more users are concerned about data privacy, the more they devalue the benefits of data-collecting technologies. This means that in the mHealth domain, benefits (eg, financial gains as in the vignette) tend to be a less compelling argument to use this technology for those who are concerned about data privacy. However, if this relationship holds for less tangible health benefits, such as more efficient treatment, better communication with medical institutions, or early detection of diseases, remains to be seen in future studies.

Finally, social norms, that is, the opinions, experiences, and recommendations of close relatives, are also influencing the intention to use mHealth apps. In fact, social norms were the strongest drivers for the intention to use mHealth technology (path coefficient=0.478) in our study. This conforms with findings from social psychological research suggesting that people tend to adopt the opinion of their peers or relatives [71]. Thus, if the social environment supports mHealth technology use, these technologies are more likely to be used.

### Implications

Based on the results, there are several possibilities for health care providers to increase the intention to use mHealth apps. First, users’ perceived data autonomy could be increased by offering an easy-to-use digital infrastructure for managing personal health data, which may ultimately increase users’ intention to use the mHealth technology. Second, because users, who are concerned about data privacy, may not want to use mHealth apps (even if they benefit them), providers may want to consider new and user-friendly ways to inform about data storage and processing policies to increase trust in critical users. This could be implemented, for example, through a user-centered app design, an easy-to-comprehend text design, and a focus on transparency [40]. Finally, to increase uptake, social norms may be activated, for instance, via testimonials of satisfied users and a reward program for recommending the app to friends and family. Additionally, customer journeys may be tracked to understand and support the social dynamics underlying the use of mHealth apps during the postpurchase phase (eg, by tracking customers’ reviews, recommendations, and posts on social media) to improve the product and ultimately increase the intention to use it [72,73].

### Limitations and Future Directions

This study has several limitations that must be addressed in future research. The model was tested on a German population. However, it is evident that the use of data-collecting technology and its acceptance are strongly influenced by culture [74]. Compared with other European countries, Germans are particularly careful when it comes to using personal information online [75]. Furthermore, the sample is homogeneous in that every person residing in Germany is required to have health insurance. Thus, the probability of using a health insurance app is significantly higher than for other mHealth apps. This may also be a reason for the high explained variance (*R*^2^) of the model. Future studies should check the validity and generalizability across different cultural backgrounds.

There is also the limitation that the sample was relatively tech-savvy, as evidenced by the proportion of participants who reported using wearables (73/250, 29.2%), which is higher than in previous studies. For instance, in 2021, only 21% of a representative German sample reported to use wearables regularly in a survey study [76], which could raise doubts about the representativeness of the presented data. By contrast, the number of wearable users may have also increased during the COVID-19 pandemic, which generally boosted digitalization in health care [77]. Nonetheless, future studies should validate our results in representative samples.

Another limitation is that the study’s scenario involves an app from a widely known German health insurance company, which generally has a very high reputation in the German health care system and whose motivation for publishing an app may be less driven by economic concerns than that of companies in the private sector. It is thus likely that participants perceived health insurance more positively than a commercial provider of mHealth apps. Follow-up studies must show whether the model we presented also explains the usage intention of commercial mHealth apps. Further, denial of coverage is a rather unlikely scenario in the German health care system. A more realistic scenario should be used in a future study.

Hence, future research should investigate which features trigger perceived data autonomy in users to shed more light on why apps are perceived as more or less trustworthy. A mixed methods approach (eg, an interview study to generate hypotheses and a subsequent survey study to validate them) would be a first step in examining the factors influencing the effects of perceived data autonomy on the intention to (not) use mHealth apps [78].

Finally, in this study, injunctive social norms were operationalized with respect to recommendations and approval of mHealth apps by friends and families. To what extent health professionals activate injunctive social norms to increase or decrease intention to use [24] remains to be seen in future studies.

### Conclusions

We showed that our model can explain the intention to use mHealth apps more effectively than previous privacy calculus models in the mHealth domain. Specifically, we were able to show that in addition to the factors related to costs and benefits included in the original privacy calculus model, the intention to use mHealth apps is influenced by 3 additional factors: (1) The perceived data autonomy has an indirect influence on the intention to use mHealth apps by reducing privacy concerns and increasing trust in the provider. (2) The trait-factor attitude to privacy explains users’ trust in the provider and shows that users who are concerned about data privacy can hardly be convinced to use mHealth apps based on their potential benefits. (3) Social norms, that is, the opinions, experiences, and recommendations shared by one’s relatives and friends, influence users’ intention to (not) use mHealth apps. Together, these findings allow health care providers to improve their products and to increase usage by targeting specific user groups.

## Appendix

Multimedia Appendix 1 Case vignette.

Multimedia Appendix 2 Questionnaire.

## Abbreviations

AP: attitude to privacy
AVE: average variance extracted
CB-SEM: covariance-based structural equation modeling
CFI: comparative fit index
CON: perceived control over personal data
CR: composite reliability
GDPR: General Data Protection Regulation
HIPAA: Health Insurance Portability and Accountability Act
IU: intention to use
mHealth: mobile health
PB: perceived benefits
PC: privacy concerns
RMSEA: root-mean-square error of approximation
SN: social norm
SNS: social network site
TP: trust in the provider
