# Supplementary material for: Extending the Privacy Calculus to the mHealth Domain: Survey Study on the Intention to Use mHealth Apps in Germany
Source: JMIR Hum Factors. 2023 Aug 16;10:e45503. doi: 10.2196/45503 (PMC10468710; doi:10.2196/45503)
Supplement: Multimedia Appendix 1 [file humanfactors_v10i1e45503_app1.docx]

### Appendix A

Your friend Alex has recently started using the app from his health insurance company and is participating in the bonus program through it. To do this, he always wears a fitness tracker with which his health and location data is recorded and processed in the app. If Alex does sport regularly, the health insurance company can pay premiums of up to € 100. In the US it is already compulsory for members of some insurance companies to wear a fitness tracker. The reimbursement of treatments is made dependent on health and fitness data.
