# Supplementary material for: Extending the Privacy Calculus to the mHealth Domain: Survey Study on the Intention to Use mHealth Apps in Germany
Source: JMIR Hum Factors. 2023 Aug 16;10:e45503. doi: 10.2196/45503 (PMC10468710; doi:10.2196/45503)
Supplement: Multimedia Appendix 2 [file humanfactors_v10i1e45503_app2.docx]

### Appendix B

| **Item** | **Item text** | **Reference** |
| --- | --- | --- |
| AP01 | Compared to other issues that concern me, privacy is not very important to me. (Reversed) | Self-constructed |
| AP02 | I do not feel very concerned about my privacy online. (Reversed) | Self-constructed |
| CON01 | I believe I can control my personal data provided to the app. | Xu et al., 2013 [14] |
| CON02 | I believe I don't have control over who can get access to my personal data collected by the app. (Reversed) | Xu et al., 2013 [14] |
| CON03 | I think I don't have control over what personal information is released by the provider of the app. (Reversed) | Xu et al., 2013 [14] |
| CON04 | I believe I have control over how personal data is used by the provider of the app. | Xu et al., 2013 [14] |
| CON06 | Privacy settings allow me to have full control over the data I provide. | Krasnova et al., 2010 [16] |
| IU01 | I should use this app as soon as possible. | Miltgen et al., 2013 [59] |
| IU02 | I would use this app. | Self-constructed |
| IU04 | I would hesitate to use this app. (Reversed) | Self-constructed |
| PB01 | Providing my personal data to the smartphone app will not entail benefits. (Reversed) | Kehr et al., 2015 [53] |
| PB03 | I believe that as a result of my personal data disclosure, I will benefit from a better, more customized service. | Kehr et al., 2015 [53] |
| PB04 | I feel that disclosing my data to the smartphone app is useful. | Sun et al., 2015 [27] |
| PB05 | Disclosing my health data to the smartphone app can make my lifestyle more productive. | Sun et al., 2015 [27] |
| PB06 | Disclosing my health data to the smartphone app can simplify my lifestyle and accelerate processes. | Sun et al., 2015 [27] |
| PB07 | The usage of the app can lead me to learn new things or think about things in new ways. | Dienlin & Metzger, 2016 [60] |
| PC02 | I am concerned about submitting data to the app, because it could be used in a way I did not foresee. | Min & Kim, 2015 [61] |
| PC07 | There would be high potential for privacy loss associated with giving personal data to the app. | Xu et al., 2013 [14] |
| PC08 | Personal data could be inappropriately used by the app. | Xu et al., 2013 [14] |
| PC09 | Providing the app with my personal data would involve many unexpected problems. | Xu et al., 2013 [14] |
| PC10 | It would be risky to give personal data to the smartphone app. | Kehr et al., 2015 [53] |
| SN01 | I feel that most people who are important to me think I should not use the app. (Reversed) | Kim & Min, 2013 [42] |
| SN02 | I feel that people who influence my behavior think that I should not use the app. (Reversed) | Kim & Min, 2013 [42] |
| SN03 | I feel that I should use the app because everybody else seems to be using it. | Kim & Min, 2013 [42] |
| SN04 | If people I value, recommend me to use this app, I would. | Self-constructed |
| SN05 | If my friends were using this app, so would I. | Self-constructed |
| TP01 | The provider of the app would be trustworthy in handling the data. | Malhotra et al., 2004 [62] |
| TP02 | The provider of the app would tell the truth and fulfill promises related to the data provided by me. | Malhotra et al., 2004 [62] |
| TP03 | I trust that the provider of the app would keep my best interests in mind when dealing with the data. | Malhotra et al., 2004 [62] |
| TP07 | I would trust the app. | Miltgen et al., 2013 [59] |
